# Extraction of the Uterus in an Effort to Remove the Placenta, and Consequent Trial for Mal-Practice

**Published:** 1845-07-01

**Authors:** 


### EDITORIAL, MEDICAL INTELLIGENCE, &c.

*Extraction of the Uterus in an effort to remove the Placenta, and consequent trial for mal-practice.—Remarks on legal responsibility for mal-practice.*—The Boston Medical Journal for April 30th ult. quotes from an English Journal an account of a practitioner in Norfork, Eng. who, in attempting to remove the after-birth in an hour or two after delivery, extracted both placenta and uterus, together with most of the large intestine. The woman died in half an hour. A post-mortem examination was held before a coroner's jury, and a verdict of manslaughter rendered. On a subsequent trial, however, an acquittal was instantly returned on the ground that the accident was caused by an error of judgment, and "*that a medical man should not be punished for the death of a patient, when he had used all the skill he possessed, and had not shown criminal neglect, or wilful inattention.*"

That such an accident should occur to any respectable practitioner, will, we presume, strike our readers with surprise, and that such a verdict was rendered, will, perhaps, be hardly less surprising. This, however, is not the first event of the kind that has happened. In fact, a late number of the Lancet gives a detailed account of a case in which the uterus was first inverted, and after long and fruitless efforts to tear it away by manual force, it was cut away with a pair of scissors; a midwife occasionally relieving the accoucheur when his fingers were fatigued ! Two cases, at least, have been reported in this country within the last few years. In one of these the practitioner was a man of considerable experience, and was reputed to be a respectable member of the profession.—The other, we believe, was an irregular practitioner. In both cases trials were had, but we are not able to refer to, or recollect the circumstances attending them, or their results. We much doubt, however, whether the principle upon which the English practitioner was acquitted, obtains in this country to the same extent in criminal or other prosecutions for mal-practice.

---

The above case furnishes a text for some remarks on the extent to which a practitioner is equitably responsible for mal-practice. The subject, however, involves numerous considerations which would require not a little space for their full discussion. We can only indulge in a few brief reflections, which will not relate to the above case in particular, but to the subject in general.

Assuming that a practitioner acts with no malicious intent, but, on the contrary, with a desire to do service to his patient to the best of his ability, and that he is faithful and attentive in his services, it is certainly extremely difficult, if not quite impossible, to determine where to draw the line of responsibility, if injury arise from errors of judgment. A superior intellect, the soundest judgment, and pre-eminent attainments, by no means afford an infallible security against serious mistakes. For an example, it has occurred to more than one distinguished surgeon to perform lithotomy when no stone in the bladder existed. Suppose the patient in such a case to die from the operation? Is the surgeon guilty of man-slaughter? Numerous analagous instances might be quoted; and we are much mistaken if in the experience of the most eminent practical members of our profession, there have not occurred not a few cases in which unfortunate results have developed errors, which, if it were possible, they would be glad to redeem, at whatever cost. We make this statement in all frankness, for we believe it to be true, and at the same time reflecting no disgrace upon the profession. It would be easy to show that from the very nature of our duties, and the subjects which they involve, this must necessarily be true.

The question then recurs, where shall we fix the limits separating venial errors from those to which a legal penalty should be rigorously attached? The more we reflect upon this question with candor and impartiality, the more difficult becomes the answer, and we much doubt if any principle can be adopted which will admit of easy application in the adjudication of cases of alleged mal-practice.

But there are other considerations involved than those which belong to the subject thus abstractedly presented.

In claiming damages, or the enforcement of penalties for mal-practice, the ground is of course taken that members of society have a right to require in medical practitioners a certain amount of knowledge and skill.—Now, has society this right, and, if so, to what extent? If such a right in reality exists, it is obvious it must have been acquired in some way or other; it cannot exist as a matter of course, inherent in the very nature of things. Now what has society done to have become possessed of a right to make a requisition upon the medical profession for a certain amount of attainment and practical ability, and if these are absent, to enforce criminal or civil penalties? Does society assume the prerogative to educate medical men? Not at all. All that society does, is occasionally to make through her legislators some small appropriations for medical schools, but not more, nor so much as to institutions for other branches of learning, and for the advantages of these the medical student is required to pay a proper pecuniary equivalent. Does society exert authority to render the profession in itself desirable and honorable? No. Society vouchsafes no legal privileges, gives no protection,—but on the contrary, has decided virtually, that a thorough medical education constitutes not the least claim to distinction or peculiar rights. Does society bestow with a liberal hand pecuniary emolument? By no means. There is not a class of men more poorly paid for their services than the members of the medical profession. How is it, then, that society presumes to act as if physicians were so deeply in its debt, or that it has such extraordinary claims, that an error of judgment, and even ignorance, are to be held to a stringent accountability! We do not hesitate to affirm that the position which society arrogates, is unjust and unwarrantable. No doubt it is very desirable that the lives and bodies of its members should be protected against mal-practice, but before society assumes the right to punish ignorance and unskilfulness, let something be done to substantiate a valid claim to the advantages of learning and skill. Let liberal appropriations be made for the promotion of medical education; let the avenues to knowledge be thrown open, successful attainment encouraged and rewarded, and the possession of a medical diploma made to confer honor on its possessor; let the profession receive protection in its just rights and dign ty; legalize the study of Anatomy; endow Hospitals, and constitute them schools of practical medicine. Let Society perform on her part these just and reasonable obligations, and then she may with some show of propriety demand on the part of the profession that its members shall not only be attentive and careful, but intelligent and well educated.

The present liability of medical men to vexatious prosecutions for mal-practice is peculiarly aggravating. In addition to the intrinsic injustice of the thing, they have to contend against the unprincipled malignity and sordid motives of any who choose to prosecute, and often against the ignorance and prejudices of those who are to adjudicate. We venture to say that a high minded honorable man never commenced a suit for mal-practice. Such an instance certainly never came within our knowledge. Those who seek this method of redress are generally of that class who would never repay with gratitude or pecuniary remuneration medical services, however important they may have been to their welfare. It is a cruel hardship for a medical man to be obliged to contend with such individuals.

Then, again, his cause is committed to the hands of a jury, who, it may be safely assumed, are incompetent to decide upon its equitable merits. This jury will most likely embrace some individuals whose feelings have been rendered hostile to the medical profession through some of the various means, which, for selfish purposes, are constantly employed to bring about such an opposition in the minds of the community. Is it just or proper that the professional duties of a medical practitioner should be decided upon by men of this stamp? Yet it can hardly be avoided in the indiscriminate selection of the members of a jury. But granting that their judgments are not in any instances liable to be affected by preconceived opposition or dislike, are jury-men competent to determine whether an error or fault in a medical practitioner be excusable or not? To do this presupposes an intimate acquaintance with the subjects involved, of which they are generally totally ignorant. In fact, we might, with exceptions and qualifications, extend these remarks to embrace the whole court as well as the jury-box; and, if our space permitted, it would be easy to adduce instances in which the instructions of Judges to Juries with reference to their verdict, fully sustain, if not the first, certainly the second ground of incompetency which has just been referred to. But it is quite needless in addressing the Medical Profession to labor to prove the incompetency of ordinary legal tribunals to adjudicate equitably upon cases of alleged mal-practice. The profession well know this to be the case, and abundant facts might be collected in which the results demonstrate it, without the necessity of argument to show why it must necessarily be so.

The conditions which society imposes upon the practice of medicine are peculiarly proscriptive and inconsistent. Other callings of anolagous responsibilities are comparatively exempt from them. Suppose, for example, an architect to construct a public edifice designed to accommodate a certain nnmber of persons ; the principles of construction prove to be defective, and through his want of judgment or ignorance, a multitude of lives are lost, injuries inflicted, &c. Is the architect in this instance held to a strict accountability ? Was there ever an instance in which punishment was inflicted for this sort of mal-practice ? Other illustrations could be cited ; but this will suffice. Now compare the actual responsibility in the two cases. The architect in his constructions has every element at his disposal. The strength of material, the principles of mechanics, &c., can all be mathematically determined and applied. Hence, there can be comparatively no excuse for error of judgment or ignorance. How different the problems which are to be solved in medical practice, where the elements are ever varying, rarely if ever to be estimated with mathematical precision, and where the result is constantly liable to be affected by contingencies which cannot be foreseen and provided against !

The truth is, society is guilty of a grievous wrong toward the Medical Profession in this particular ; and this is one of the many instances exemplifying the fact that the duties and responsibilities of the public toward the profession, are, as it is most charitable to think, very imperfectly understood, and, consequently, very imperfectly performed. Society avails itself of the benefits which medical science confers upon it, but refuses a grateful reciprocation. Its relations have become too much those of a tyrannical task-master, rather than those of a fostering and indulgent parent. In thus expressing ourselves with some warmth, it may be proper to say, that our feelings are not sharpened by any thing derived from our own experience, but have been derived from general observation and reflection upon the subjeet. If it should appear to any of our readers that our assertions are too strong, or our positions untenable, we shall be glad to have them corrected, and we will willingly afford reasonable space for the expression of different views, if there be any difference of opinion on the subject among the members of the profession. To our mind, the position of a medical man, especially if he practice Surgery, (which is more exposed because the results are more palpable,) is invested with a risk to which it ought not to be exposed. His reputation, which is his all, and should be dear to him as life, is at the mercy of events which he cannot secure himself against. No one, whatever be his character and attainments, is perfectly safe. Public opinion demands that he shall not decline any responsibility, and yet by assuming it, he may incur the danger of becoming the victim of personal malice or cupidity, which invoke successfully in their behalf the tribunals of Justice !

But we may be asked would you have no personal accountability ; would you remove all penalties and liabilities ? We answer, no. We would have a substitute for the present method of repressing ignorant assumption, recklessness and neglect, which would involve less liability of personal injustice, and at the same time be more effectual in accomplishing the object, which is, or should be, to prevent the evils by proper penalties. We are not prepared, if we had space, to present any matured plan. The great principle, however, of the reform which we would advocate, is that the adjudication and presentment of cases of mal-practice should be made by the medical profession itself, and the penalties, excepting in extraordinary cases, should be reprimand, suspension, or expulsion from the Profession with subsequent disability to practice. For most cases these would afford punishment sufficiently adequate to the fault. It would do away with the sordid inducement now held out to unprincipled individuals, to endeavor to speculate upon the ill success, or, it may be, the errors of the medical attendant. Most suits, are, as we all know, dictated by these motives. The details of such a proposed improvement we must for the present forego; they are connected with other matters relating to medical reform, upon which action should be had simultaneously. That any thing can be effected at present is not to be expected, but we trust that the time is not distant, when such a re-organization of the Profession will be accomplished, that by the union of law, public opinion and the sentiments of the profession, the practice of medicine will be rendered more useful to Society, and more satisfactory to the practitioner. In the mean time, reflection, and free discussion, will expedite and prepare the way for future definitive action.

---

In offering the foregoing remarks at a time when a trial for malpractice which has excited much interest both in and out of the profession has recently taken place in this city, we deem it proper to add, that they have no intentional reference to, or connection with this occurrence. They were in fact, written some weeks before this trial took place. We deem it proper to state this, because it might otherwise be supposed that we designed some allusion to circumstances or persons involved in this trial, which, especially as the jury in the case referred to were unable to agree, and another trial will probably be had, would be manifestly improper. We trust that there will be no misapprehension on this head. What we have said relates to the general principle upon which all trials of this kind at the present time are based. We find fault with, and complain of the principle itself, without, on this occasion, going into any particular application of it. In the few remarks which we have made, we have contemplated the subject as it affects us, that is the medical profession. But if we were to take another point of view, regarding it from an opposite position—viz—that of Society, or the Public, we should find that the evil results are not limited to the medical profession. Society, in this matter, is not only guilty of injustice to the profession, but is unjust to itself, and faithless to its own interests. The considerations belonging to this view, should also engage the attention of medical men, and through them of the community, but we have already extended this article to such length that we must reserve farther remarks for some future occasion.

